# Enhancement of Motor Function Recovery after Spinal Cord Injury in Mice by Delivery of Brain-Derived Neurotrophic Factor mRNA

**DOI:** 10.1016/j.omtn.2019.06.016

**Published:** 2019-06-29

**Authors:** Samuel T. Crowley, Yuta Fukushima, Satoshi Uchida, Kazunori Kataoka, Keiji Itaka

**Affiliations:** 1 Department of Biofunction Research, Institute of Biomaterials and Bioengineering, Tokyo Medical and Dental University (TMDU), 2-3-10 Kanda-Surugadai, Chiyoda-Ku, Tokyo 101-0062, Japan; 2 Innovation Center of Nanomedicine (iCONM), Kawasaki Institute of Industrial Promotion, 3-25-14 Tonomachi, Kawasaki-Ku, Kawasaki-Shi 210-0821, Japan; 3 Department of Bioengineering, Graduate School of Engineering, The University of Tokyo, Bunkyo-ku, Tokyo 113-8656, Japan; 4 Policy Alternatives Research Institute, The University of Tokyo, 7-3-1 Hongo, Bunkyo-ku, Tokyo 113-0033, Japan

## Abstract

Spinal cord injury (SCI) is a debilitating condition that can cause impaired motor function or full paralysis. In the days to weeks following the initial mechanical injury to the spinal cord, inflammation and apoptosis can cause additional damage to the injured tissues. This secondary injury impairs recovery. Brain-derived neurotrophic factor is a secreted protein that has been shown to improve a variety of neurological conditions, including SCI, by promoting neuron survival and synaptic plasticity. This study treated a mouse model of contusion SCI using a single dose of brain-derived neurotrophic factor (BDNF) mRNA nanomicelles prepared with polyethylene glycol polyamino acid block copolymer directly injected into the injured tissue. BDNF levels in the injured spinal cord tissue were approximately doubled by mRNA treatment. Motor function was monitored using the Basso Mouse Scale and Noldus CatWalk Automated Gait Analysis System for 6 weeks post-injury. BDNF-treated mice showed improved motor function recovery, demonstrating the feasibility of mRNA delivery to treat SCI.

## Introduction

Spinal cord injury (SCI) affects approximately 300,000 people per year worldwide. Young men make up the majority of cases, usually caused by automobile accidents, falls, sports injuries, and violence.^1^, ^2^ The physical and financial effects of SCI are severe, with lifetime healthcare and indirect costs typically reaching into millions of dollars per patient.^3^

Recovery from SCI is made difficult by the physiological changes and loss of neurons following the initial injury. In the days to weeks following SCI, the injured tissue suffers from inflammation and apoptosis, loss of myelin, and formation of a glial scar that prevents new axon growth. This secondary injury impairs motor function recovery. Methods to prevent the secondary injury might improve long-term recovery by keeping the neural tissue alive during this critical period.^4^

One such method may be brain-derived neurotrophic factor (BDNF), a secreted protein that binds to the tropomyosin receptor kinase B (TRKB) receptor on neurons, activating the mitogen-activated protein kinase-extracellular signal-related kinase (MEK-ERK), protein kinase B (AKT), and phospholipase Cγ1 (PLCγ1) pathways, which promote neuron survival and synaptic plasticity. However, delivery of BDNF protein is a formidable challenge. BDNF does not cross the blood-brain barrier, necessitating direct injection into the CNS. Its short half-life and poor tissue penetration make multiple injections or continuous infusion necessary.^5^, ^6^, ^7^

Gene therapy offers potential advantages for BDNF delivery. If *BDNF* DNA can be delivered to the injured tissue, BDNF could be produced directly where it’s needed without multiple doses or intrathecal infusion. In fact, previous research has attempted *BDNF* gene therapy to treat SCI.^8^, ^9^, ^10^ Others have combined *BDNF* gene therapy with cell transplantation^11^, ^12^ or a combination of gene therapy, cell transplantation, and multi-channeled implants to guide axon growth.^13^, ^14^

Recent years have seen a growth in interest in mRNA as a therapeutic molecule for transient gene therapy. mRNA has some advantages over DNA; most importantly, mRNA does not need to enter the nucleus to produce protein, allowing it to transfect a larger portion of cells,^15^ and mRNA is capable of producing greater amounts of protein than DNA.^16^ Plasmid DNA has a small chance of integrating into the host genome, causing potentially dangerous mutations, but mRNA is not capable of genomic integration. While mRNA is only able to produce protein for a limited amount of time, many DNA delivery studies show similarly short expression. This limited duration may be advantageous, as some proteins may be dangerous when overexpressed for long periods; for example, BDNF has been implicated in chronic pain.^17^, ^18^, ^19^

mRNA delivery may also have advantages over cell transplantation. Transplanted cells typically do not become functional neurons in the spinal cord, but they do secrete useful paracrine factors that can support surviving neural tissue. mRNA delivery could produce these factors using the body’s own cells, avoiding complicated or potentially risky cell implantation.^20^, ^21^, ^22^ mRNA is also capable of delivering membrane-bound or intracellular proteins that transplanted cells cannot secrete.

**Figure 1:**
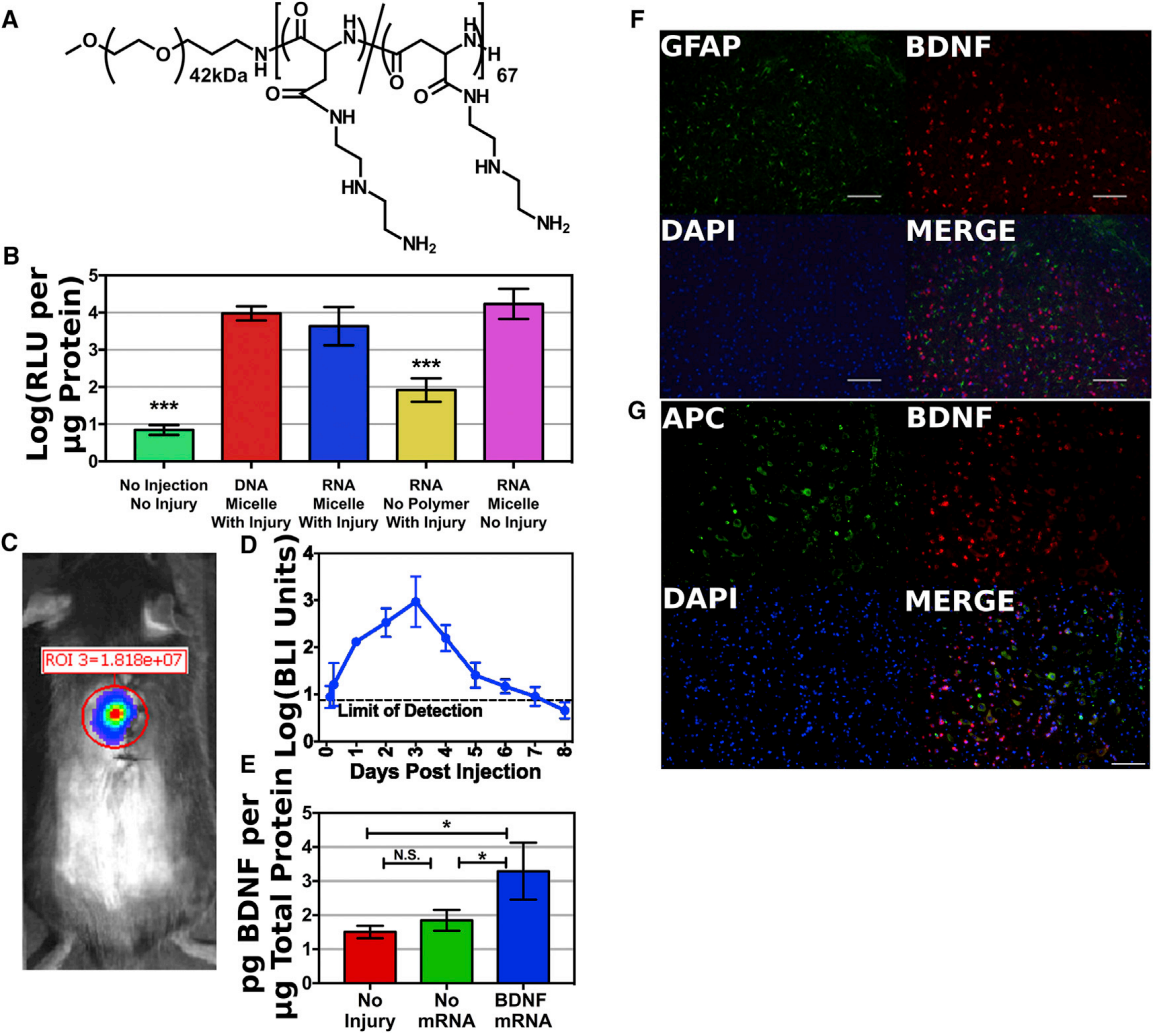
Delivery of mRNA Nanomicelles to Spinal Cord mRNA nanomicelles were formed by mixing mRNA with PEG-PAsp(DET), shown in (A). At physiological pH, one of the two amino groups in the DET side chain is protonated and positively charged, allowing binding to the anionic phosphate backbone of the mRNA. The second amino group of the DET side chain is protonated during endosomal acidification, increasing local charge density, which is believed to assist in endosomal disruption and mRNA release into the cytoplasm.^24^, ^26^*Firefly luciferase* mRNA and DNA nanomicelles were prepared, and 500-ng doses of nucleic acid were injected into mouse spinal cords. Luciferase activity in homogenized tissue at 48 h post-injection was measured in (B) (n = 5). Luciferase activity was measured over time using bioluminescent imaging in (C) and (D) (n = 6). *BDNF* mRNA nanomicelles were prepared and injected into injured mouse spinal cords at 500-ng doses of mRNA. Spinal cord tissues were collected at 48 h post-injection and homogenized. ELISA was performed to measure BDNF concentrations relative to total protein concentration in (E) (n = 3). All error bars represent sample SD. (F and G) Spinal cord tissue sections were stained for BDNF and GFAP to identify astrocytes in (F) and APC to identify oligodendrocytes in (G).

However, mRNA has disadvantages as well. The biggest disadvantage is its vulnerability to nuclease digestion. To help protect the mRNA against digestion, it is often mixed with cationic polymers to form nanoparticles. PEG-b-poly{N-[N-(2-aminoethyl)-2-aminoethyl]aspartamide} (PEG-PAsp(DET))^23^, ^24^ (Figure 1A) is one such cationic polymer. While it was originally designed for use with DNA, it is also useful with mRNA.^25^ The 1,2-diaminoethane side chain undergoes additional protonation upon endosomal acidification to form a diprotonated structure, which promotes membrane disruption and endosomal escape.^26^ When negatively charged RNA and positively charged PEG-PAsp(DET) are mixed together, they form mRNA nanomicelles. This mRNA nanomicelle formulation has been used to deliver mRNA to several organs, including the liver,^15^ knee joint,^27^ olfactory nerves,^28^ and brain.^25^, ^29^

This study sought to apply PEG-PAsp(DET) *BDNF* mRNA nanomicelles to treat SCI. Mice were given contusion SCI and immediately injected with single 500-ng doses of mRNA loaded in nanomicelles. Motor function was monitored for 6 weeks post-injury, and *BDNF* mRNA-treated mice showed significantly better motor function recovery than their non-treated counterparts. As far as the authors know, this is the first study to deliver mRNA to the spinal cord and the first study to attempt to use mRNA to treat SCI.

## Results

### mRNA Synthesis

Bioanalyzer analysis of the *BDNF* and *FLuc* mRNA indicates that both mRNAs were successfully produced (Figure S1).

### Protein Expression after mRNA Nanomicelle Injection

All animal experiments were approved by the Institutional Animal Care and Use Committee (IACUC) of the Innovation Center of NanoMedicine, Kawasaki Institute of Industrial Promotion. To test for protein expression after mRNA nanomicelle injection, mice were given intraspinal injections of 500-ng doses of *firefly luciferase* mRNA loaded in nanomicelles. To see the influence of SCI in luciferase expression, *luciferase* mRNA was injected into injured tissue immediately after contusion SCI. Expression in injured tissue was measured using a homogenized tissue assay at 48 h post-injection (Figure 1B). mRNA nanomicelles were compared to naked mRNA and to DNA nanomicelles. Naked mRNA produced significantly weaker luciferase activity than either nanomicelle formulation, but there was no significant difference between RNA and DNA. Injured and uninjured mice were compared using mRNA nanomicelles. No significant difference between injured and uninjured mice was observed.

Luciferase activity over time was determined by bioluminescent imaging in non-injured tissue (Figures 1C and 1D). Bioluminescence peaked at approximately 72 h post-injection, and it was approximately 3 orders of magnitude greater than the limit of detection. Luminescence was located near the injection site, indicating that the mRNA nanomicelles did not travel far from the injection site.

Therapeutic protein expression was tested using *BDNF* mRNA nanomicelles. Mice were given contusion SCI with or without 500-ng doses of *BDNF* mRNA loaded in nanomicelles. Mice were sacrificed at 48 h post-injury and spinal cord tissues were collected and homogenized. BDNF concentrations were determined by ELISA and expressed as picograms BDNF per microgram total protein (Figure 1E). There was no significant change in BDNF expression following SCI without mRNA injection. The BDNF concentration in mRNA nanomicelle-treated mice was approximately twice that of the non-injured and non-injected mice, a statistically significant increase (p < 0.05).

To identify the cells transfected by the PEG-PAsp(DET) mRNA nanomicelles, mice were given contusion SCI and 500-ng doses of *BDNF* mRNA loaded in nanomicelles. At 48 h post-injection, spinal cords were removed and sectioned. Spinal cord tissue sections were stained for glial fibrillary acidic protein (GFAP) to identify astrocytes (Figure 1F) or for allophycocyanin (APC) to identify oligodendrocytes (Figure 1G), as well as for BDNF. However, both stains showed only partial overlap of GFAP or APC with BDNF, suggesting that more than one cell type may be transfected by the nanomicelles.

An additional measurement of BDNF expression was carried out by giving mice contusion SCI and 500-ng doses of *BDNF* mRNA or *BDNF* DNA loaded in nanomicelles. At 24 h post-injection, spinal cords were removed and sectioned. Spinal cord tissue sections were stained for BDNF and immunofluorescence was quantified (Figure S3). In this experiment, both mRNA and DNA nanomicelles produced significantly higher protein expression than non-treated mice, but the DNA nanomicelles produced more protein than the mRNA nanomicelles.

### Motor Function Assessment by Basso Mouse Scale

Mouse motor function was estimated by visual observation according to the Basso Mouse Scale (BMS) system.^30^ Mice were allowed to walk in an open field, and the observer took notes on several aspects of the animal’s gait. The BMS score ranges from 0 to 9 and is determined according to a flowchart. The BMS subscore has a range from 0 to 11. All healthy mice have a score of 9 and subscore of 11, while a score or subscore of 0 represents total hindlimb paralysis. BMS score and subscore were plotted over time (Figures S4A and S4B). *BDNF* mRNA-treated mice appeared to have better BMS scores and subscores, however, neither score showed a statistically significant difference between mRNA-treated mice and untreated mice. None of the injured mice obtained a full score of 9 or a full subscore of 11, because none of the mice were able to achieve normal torso stability. This result led us to perform a more quantitative assay to clarify the apparent difference between *BDNF*-treated and non-treated mice.

### CatWalk Measurement Results

**Figure 2:**
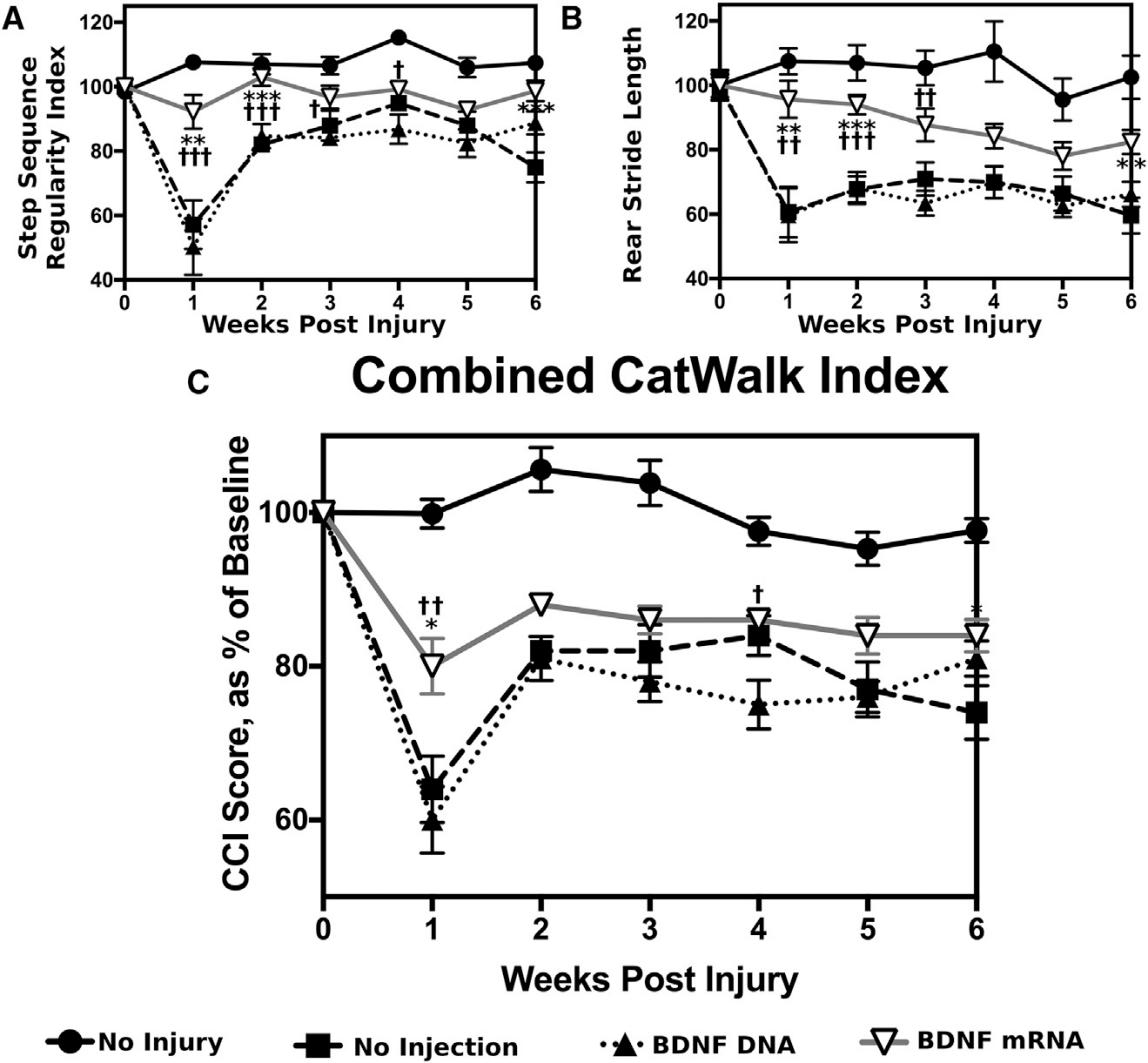
Selected Results from the CatWalk Analysis The entire CatWalk data are presented in Figures S5–S10. Pre-injury baseline measurements were collected prior to injury, and post-injury measurements were taken every week for 6 weeks following injury. Data are presented relative to pre-injury baseline. The step sequence regularity index in (A) is a measure of coordination. Rear stride length in (B) is how far the mouse moves its hindlimbs between steps. CatWalk data were used to calculate the combined CatWalk index (CCI) scores, according to a previously published method,^32^ in (C). Statistical significance was determined using one-way ANOVA followed by Tukey’s test for multiple comparisons. Statistically significant differences between the *BDNF* mRNA-treated mice and non-treated mice are reported using asterisks (*), while statistically significant differences between the BDNF mRNA-treated mice and BDNF DNA-treated mice are reported using daggers (**†**). All error bars represent SEM. *p < 0.05, **p < 0.01, ***p < 0.001; n = 12.

Gait analyses were performed using the CatWalk Automated Gait Analysis System, which provides quantitative information about speed, step regularity, stride length, step intensity, etc.^31^ Mice were monitored on the CatWalk every week for 6 weeks after injury and injection. Videos were classified and data were analyzed using custom software. Two CatWalk parameters were chosen to represent the results (Figure 2). The entire set of CatWalk data are presented in Figures S5–S11.

*BDNF* mRNA-treated mice showed significantly better step sequence regularity index than non-treated or DNA-treated mice (Figure 2A), indicating better coordination at weeks 1 and 2. mRNA-treated mice also showed significantly better rear stride length than non-treated or DNA-treated mice at weeks 1 and 2 (Figure 2B), indicating a more normal gait.

In general, the CatWalk parameters presented in Figure 2 and several of the parameters in Figures S5–S11 show that the *BDNF* mRNA-treated mice had statistically significantly better scores than the non-treated controls and/or DNA-treated mice. These differences usually appeared in weeks 1 and 2 of the study, implying that the *BDNF*-treated mice have a more rapid recovery of motor function and a more normal gait than their non-treated counterparts.

### Combined CatWalk Index

A mathematical model to combine all CatWalk data into a single score^32^ was used to provide additional analysis of the CatWalk data (Figure 2C). This method was developed by correlating BMS scores with CatWalk data from many measurements. The *BDNF* mRNA-treated mice appeared to have better combined CatWalk index (CCI) scores than their non-treated and DNA-treated counterparts in week 1, showing a similar trend to the CatWalk data in Figures 2A and 2B.

### Luxol Fast Blue Staining

**Figure 3:**
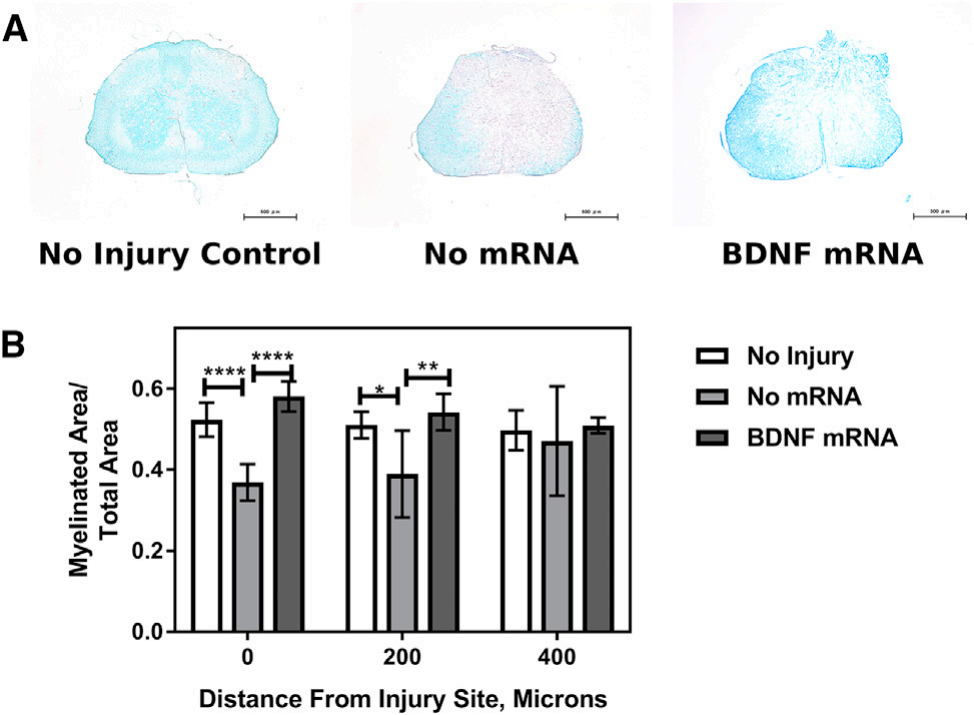
Luxol Fast Blue Staining of Spinal Cord Tissue Spinal cords were collected from mice at 2 weeks post-injury, transverse sectioned, and stained with Luxol Fast Blue, which stains myelinated tissue blue. Representative images of the non-injured control, no RNA control, and *BDNF* mRNA-treated mice are shown in (A). The ratio of myelinated to total area is plotted in (B). All error bars represent sample SD. Statistical significance was determined by one-way ANOVA followed by Tukey’s multiple comparisons test. *p < 0.05, **p < 0.01, ***p < 0.001; n = 7.

Spinal cord tissue was sectioned at 0, 200, and 400 μm from the injury site and stained with Luxol Fast Blue to stain myelinated tissues (Figure 3). Representative images at 0 μm from the injury site from the non-injured controls, non-treated controls, and *BDNF* mRNA-treated mice are shown in Figure 3A. The ratio of myelinated area to total area is plotted in Figure 3B. At 0 and 200 μm from the injury site, the *BDNF* mRNA-treated mice showed a significantly higher ratio of myelinated to total area than the no mRNA controls, suggesting that *BDNF* mRNA-treated mice have more myelin than their non-treated counterparts.

### Inflammatory Cytokine Expression

**Figure 4:**
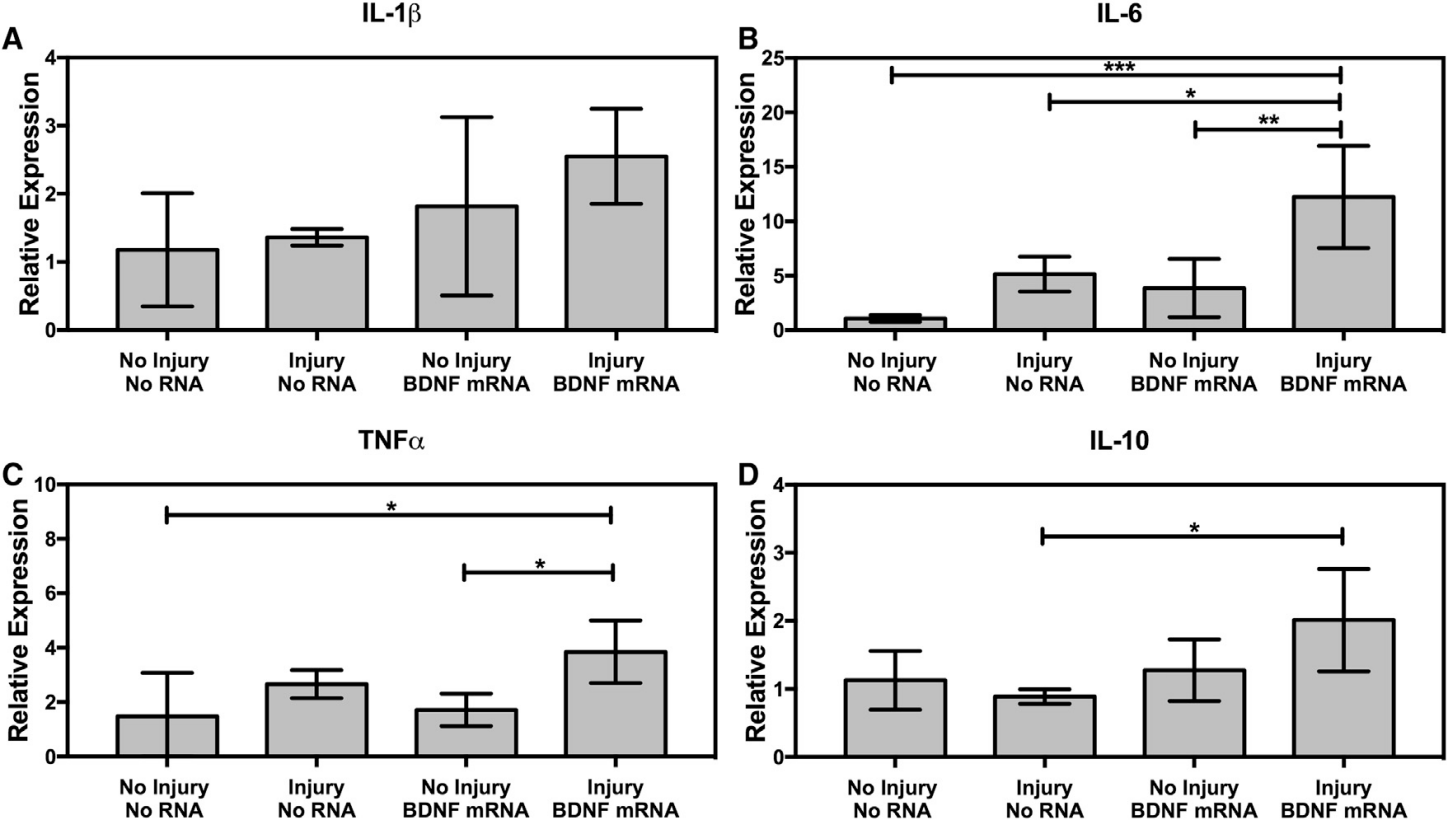
Cytokine Expression Mice were given spinal cord injuries and either no RNA or *BDNF* mRNA nanomicelles. (A–D) The relative expression levels of IL-1β (A), IL-6 (B), TNF-α (C), and IL-10 (D) are shown. Expression levels are determined relative to actin control and the non-injured no RNA group. Error bars represent sample SD. Statistical significance was determined by one-way ANOVA followed by Tukey’s multiple comparisons test. *p < 0.05, **p < 0.01, ***p < 0.001; n = 5.

Because RNA delivery can potentially trigger inflammation, inflammatory cytokine levels following SCI and mRNA injection were determined by qRT-PCR (Figure 4). The combination of contusion SCI and BDNF mRNA injection significantly increased the expressions of interleukin (IL)-6 and tumor necrosis factor alpha (TNF-α), suggesting higher inflammation, but also showed significantly higher expression of the anti-inflammatory cytokine IL-10 compared to mice that were injured but not given mRNA. IL-4 expression was measured but not detected in any samples. However, mice that received mRNA without SCI showed no significant changes in cytokine expression.

## Discussion

A single dose of 500 ng mRNA or DNA was chosen due to limitations of particle stability and injection volume. An injection volume of 1 μL was chosen to avoid causing additional damage to the spinal cord during the injection. However, the maximum concentration at which stable nanomicelles can be formed with PEG-PAsp(DET) is 500 ng/μL (unpublished data), necessitating a maximum dose of 500 ng. A single injection was chosen because the intraspinal injection method requires surgery to expose the spinal cord, and repeated surgeries creates the risk of additional injury. The ratio of amino groups in polymers to phosphate in mRNA (N:P ratio) of 3:1 was chosen based on a previous study of mRNA nanomicelle delivery to mouse brain^29^ and a study that indicates that additional polymer beyond the N:P of 3:1 is not bound to the polyplex nanomicelle.^33^

mRNA nanomicelles formed with PEG-PAsp(DET) were shown to be able to transfect spinal cord tissue after injection directly into the spinal cord (Figure 1). Firefly luciferase activity was located near the injection site by detection with *in vivo* bioluminescent imaging (BLI) at 5 min after intraperitoneal (i.p.) injection of luciferin. BLI has been commonly applied to measure luciferase activity in the mouse brain, and luciferin is known to cross the blood-brain barrier.^34^ The 5-min delay between luciferin injection and BLI measurement is likely acceptable based on a quantitative study of BLI for detecting luciferase expression in the liver, which found that BLI signal was fairly stable over 50 min.^35^ A previous study^8^ used firefly luciferase-expressing plasmid DNA polyplexes prepared using a non-PEGylated version of the PAsp(DET) polymer, but 2 μg plasmid DNA was injected into the subdural space between the fourth and fifth lumbar vertebrae, while here we injected 500 ng mRNA into the injured spinal cord parenchyma. The previous experiment produced detectable luciferase activity along the entire length of the spinal cord, while the current experiment only produced luciferase activity around the injection site. Perhaps the reduced area in this current experiment was due to the decreased dose, intraspinal injection method, or a combination of both. The luciferase expression remained above the limit of detection for approximately 6 days. Because BDNF is a secreted protein, it might cover a wider area than firefly luciferase, which is trapped inside the cells that produce it.

mRNA and DNA nanomicelles were compared in injured tissue using a homogenized tissue assay because it provides more accurate quantification than BLI. However, no significant difference was seen between DNA and mRNA nanomicelles, nor was there a significant difference between injured and uninjured tissue. This shows that injured spinal cord tissue can still efficiently produce protein after nanomicelle injection. The importance of the nanomicelle formulation is shown by the significantly reduced luminescence of the naked mRNA control, demonstrating that the nanomicelle improves mRNA stability or uptake or both.

Immunofluorescent staining of spinal cord tissue sections was not able to identify a single cell type transfected by the PEG-PAsp(DET) mRNA nanomicelles. Instead, partial overlap between BDNF-expressing cells and GFAP- or APC-expressing cells suggests that more than one cell type is transfected. More importantly, because BDNF is a secreted protein, the type of transfected cell may not matter, as any cell should be able to produce and secrete the protein for therapeutic effect.

SCI experiments were carried out to determine if mRNA could have a potential therapeutic role. Injured mice were given 500-ng doses of *BDNF* mRNA loaded into nanomicelles and followed for 6 weeks using 2 different methods to assess motor function. The BMS method indicated that *BDNF*-treated mice appeared to have higher BMS scores and subscores than non-treated injured mice (Figure S4), indicating better motor function recovery. However, these differences were not statistically significant.

The lack of statistical significance in the BMS data may be related to several factors. The Infinite Horizons Spinal Cord Impactor is difficult to consistently control, and it usually produced peak impact forces higher than the 50 kdynes the instrument was set to, with substantial variation between mice. Additionally, injury severity can be different even when peak impact forces are similar, due to differences in impact angle, position on the spinal cord, etc. One report was able to reduce variation in severity by also controlling for tissue displacement during impact,^36^ but the authors had to exclude several mice from the study due to peak impact force or displacements that fell outside the specified window. Additionally, the BMS method is subject to human error, and observers usually require training before they can reliably use the method. To further improve the method, mice are observed by multiple people who are blinded to the experimental treatment. The BMS data collected in this experiment were collected by a single, untrained, and non-blinded observer. These factors may have contributed to the greater variation in BMS results and prevented statistical significance.

CatWalk automated gait analysis showed several measurements that indicated that *BDNF* mRNA-treated mice had statistically significantly improved motor function compared to their non-treated counterparts (Figure 2) in weeks 1 and 2. However, several other measurements indicated a slight but insignificant improvement over non-treated controls, or no change at all (Figures S6–S11).

The combined CatWalk index scores are produced from a combination of all CatWalk data, and they showed that *BDNF*-treated mice appear to have better motor function recovery than non-treated mice. This difference was statistically significant in week 1, corroborating the CatWalk data (Figure 2).

The CatWalk system has the advantage of being much more objective than the BMS, and it requires less training to use. However, there was still substantial variation between individual mice and between time points, which prompted the decision to present all CatWalk data as a percent of pre-injury baseline. Mice were accustomed to the instrument by collecting 3 pre-injury baseline measurements in the week before injury. Perhaps a more extensive training period would have reduced variation and produced more significant results. Nonetheless, statistically significant differences were found between RNA-treated mice and non-treated mice. These differences were found in measurements of movement speed, coordination, intensity, and gait. While RNA-treated mice do not show complete recovery, they do show a more normal overall gait than their non-treated counterparts at weeks 1 and 2, before the non-treated mice recover from their injuries. Mice tend to spontaneously recover from moderate SCIs,^30^ but the severity of the injuries in this experiment may have been too mild. However, if the injury level is too severe, the mice do not recover within 6 weeks, even with mRNA treatment. Controlling the severity of the injury is difficult, but it is possible that a slightly more severe injury would have created a better distinction between mRNA-treated and non-treated mice.

The combined CatWalk index system was developed by correlating BMS scores with CatWalk data, and it was motivated by the large amounts of data produced by the CatWalk system. The mathematical model was created by performing simple least-squares linear regression between the BMS data and all CatWalk parameters from several hundred measurements,^32^ so a more advanced regression model could improve the system. However, this simple system appears able to successfully summarize CatWalk data, maintain statistical significance, and be much more objective than the BMS system. The combined CatWalk index scores in this study reflect the observed CatWalk data, with *BDNF* mRNA-treated mice showing significantly better scores at week 1.

mRNA-treated mice showed earlier motor function recovery than both non-treated mice and DNA-treated mice, even though the DNA and mRNA nanomicelles showed no significant difference in the luciferase expression experiment in Figure 1. The two formulations might still have different effects while producing similar levels of protein. Because DNA must enter the nucleus to produce protein, mRNA is expected to transfect a higher number of cells. However, because DNA can create several copies of mRNA when it does enter the nucleus, each DNA-transfected cell could produce more protein than a single mRNA-transfected cell. So the DNA nanomicelles are likely to transfect a small number of highly expressing cells, while the mRNA nanomicelles are likely to transfect a large number of less highly expressing cells. Furthermore, mRNA nanomicelles have been shown to produce protein at earlier time points than DNA nanomicelles,^25^ and early intervention is likely to be important in preserving motor function following SCI.

Luxol Fast Blue staining suggests that *BDNF*-treated mice have less loss of myelin at 2 weeks post-injury, based on the images having a greater ratio of myelinated to total area than the non-treated controls (Figure 3). However, the RNA-treated tissue appears less organized, which might be explained by inserting the needle into the tissue for the mRNA nanomicelle injection. The greater myelinated-to-total area ratio in the *BDNF* mRNA-treated mice may suggest a possible mechanism for how *BDNF* mRNA protects motor function following SCI. BDNF might not just prevent a loss of myelin but might encourage the formation of myelin.^37^ This could protect the neuron pathways in the spinal cord and help maintain motor function.

Cytokine expression was measured to determine if mRNA delivery had any effect on inflammation (Figure 4). Mice that were given *BDNF* mRNA nanomicelles without SCI showed no significant changes in cytokine levels compared to the control mice, suggesting that mRNA nanomicelle injection by itself does not induce inflammation. Injured mice with mRNA nanomicelles showed higher expressions of the inflammatory cytokines IL-6 and TNF-α. However, injured RNA-treated mice also showed higher expression of the anti-inflammatory cytokine IL-10, suggesting that the pro-inflammatory TNF-α and IL-6 may be counteracted. The mRNA used in this study was normal mRNA, using the four standard nucleotides A, U, G, and C. This type of mRNA is known to cause some inflammatory responses. Chemically modified mRNA, containing pseudouridine and 5-methylcytodine, might reduce the inflammatory response,^38^ and it should be used in future studies. Nevertheless, the relative expression levels seen in this study are similar to levels seen in previous studies of SCI^8^ or mRNA delivery to the brain.^25^ Additionally the histology results do not show significant differences in the overall morphology of mRNA-treated and non-treated spinal cords, suggesting that mRNA treatment does not cause significant additional inflammation compared to SCI alone.

This study is the first known use of mRNA delivery to the spinal cord. While direct injection into the spinal cord carries risks of additional damage, in the context of traumatic SCI this risk might be acceptable. This study is likely to be more clinically relevant than previous studies. Hayakawa et al.^8^ gave mice DNA polyplexes 24 h prior to SCI, such that BDNF expression was already strong when the injury was administered. Cell or scaffold implants require invasive surgical procedures, genetically modified cells, and advanced materials that might not be available in a medical emergency. This study was able to achieve significant improvements in motor function recovery by a single dose of 500 ng mRNA. This implies that BDNF expression is important in the days immediately after injury. Extending the duration of BDNF expression may be necessary to further improve recovery. A study using inducible expression vectors showed that 3 weeks of BDNF expression was sufficient to improve outcome in SCI;^12^ however, BDNF expression from mRNA is not likely to have lasted for more than 1 week in this study, based on luciferase expression results in Figure 1. This was sufficient to produce improved motor function in the first 2 weeks after injury, but the non-treated and DNA-treated mice mostly caught up to the mRNA-treated mice by weeks 3 and 4.

Further modifications to the procedure may be able to improve results. Using more than one dose of mRNA nanomicelles would extend protein expression and could prevent further neuron death. However, every injection requires a surgery and carries the risk of additional injury. mRNA delivery allows for the easy delivery of a variety of proteins, because all mRNA sequences have essentially the same structure and nanomicelle production is essentially identical. Different proteins, or combinations of proteins, could improve motor function recovery. For example, overexpression of BDNF’s receptor, TRKB, has been shown to improve the activity of BDNF.^39^ mRNA delivery is well suited for the overexpression of non-secreted and membrane-bound proteins, because mRNA does not need to enter the nucleus to produce protein. DNA requires nuclear entry, reducing the number of transfected cells, even if total protein production is higher.

The proof of concept demonstrated in this study is promising for further research using mRNA to treat SCI. The methods developed to deliver mRNA to the spinal cord and measure motor function can be applied to study additional proteins and identify potential therapeutics.

## Materials and Methods

### *BDNF* and *FLuc* mRNA Production

DNA templates for *in vitro* transcription (IVT) of mRNA were constructed by inserting a protein-expressing fragment into a pSP73 vector (Promega, Madison, WI, USA) that included a T7 promoter. Prior to the insertion, a 120-bp poly A/T sequence was cloned into the pSP73 vector downstream of the protein-coding sequence, so that mRNA possessing a 120 adenine poly(A) tail at the 3′ terminal end could be obtained by a simple procedure of IVT from the pSP73-poly(A) vector. The protein-expressing fragments were obtained from DNAs encoding *BDNF* (pUNO1-h*BDNF*a; InvivoGen, San Diego, CA, USA) and *firefly luciferase* (pGL4; Promega).

*BDNF* template DNA was prepared by digesting 60 μg pSP73-*BDNF*-poly(A) plasmid with BsmBI at 55°C overnight, followed by blunting with T4 DNA Polymerase. Residual RNase was removed using Proteinase K digestion in 1% SDS at 60°C for 1 h. Digested DNA was purified by phenol:chloroform extraction and isopropanol precipitation. *BDNF* mRNA was prepared using the Ambion mMessage mMachine T7 Ultra kit (Life Technologies, Carlsbad, CA, USA) with ARCA 5′ Cap Analog and 1 μg template DNA. The IVT reaction was incubated at 37°C for 3 h. mRNA was purified using the spin column-based QIAGEN RNeasy Mini Kit (QIAGEN, Hilden, Germany). RNA was quantified by absorbance spectrophotometry using a Nanodrop 2000 (Thermo Fisher Scientific, Wilmington, DE, USA). RNA quality was assessed using the Agilent 2100 Bioanalyzer chip-based capillary electrophoresis system (Agilent Technologies, Santa Clara, CA, USA) and native agarose gel electrophoresis. *Firefly luciferase* mRNA was prepared using the same procedure as above but with pSP73-FLuc-Poly(A) plasmid as the transcription template. Bioanalyzer data for each mRNA are shown in Figure S1.

### mRNA Nanomicelle Preparation

*BDNF* mRNA nanomicelles were produced by mixing 37.5 μg *BDNF* mRNA in 45 μL 10 mM HEPES (pH 7.4) with 150 μg PEG-PAsp(DET) 42-67 in 30 μL 10 mM HEPES (pH 7.4) to create nanomicelles with an N:P ratio of 3:1 and an mRNA concentration of 500 ng/μL. PEG-PAsp(DET) was synthesized by the quantitative aminolysis of PEG-b-poly(β-benzyl L-aspartate) with diaminoethane, as described previously.^40^ The “42-67” nomenclature denotes a PEG molecular weight of 42 kDa and an average PAsp degree of polymerization of 67. Nanomicelles were kept on ice until they were injected into mice. *Firefly luciferase* mRNA nanomicelles and plasmid DNA nanomicelles were prepared using a similar procedure. Naked mRNA controls were produced by omitting the PEG-PAsp(DET).

### Luciferase Expression in Injured Tissue

Female C57BL/6J mice (n = 5) were anesthetized by i.p. injection of 0.3 mg/kg medetomidine HCL, 4 mg/kg midazolam, and 5 mg/kg butorphenol tartrate mixture, and their spines were surgically exposed under a surgical microscope and stabilized using clamps. Laminectomies were performed at the 11^th^ thoracic vertebra to expose the spinal cord. After the laminectomy was performed, the animal was transferred to an Infinite Horizons IH-0400 Impactor (Precision Systems and Instrumentation, Fairfax Station, VA, USA). The impactor tip was carefully aligned with the center of the exposed spinal cord surface and lowered until the tip barely touched the surface. The tip was then raised by turning the vertical adjustment knob 4 turns, approximately 5 mm above the spinal cord surface. Impacts were carried out using a peak impact force of 50 kdyne.

Immediately after injury, a 30G needle was carefully used to create an incision in the dura mater. A 500 ng/μL solution of *FLuc* mRNA loaded in nanomicelles was loaded into a glass syringe with a 30G needle. A stereotactic injection apparatus was used to hold the syringe during injection. The needle was carefully inserted into the spinal cord through the previously created dura mater incision, at an angle of 40° from vertical to a depth of 1.5 mm below the surface of the spinal cord. Mice were injected with 1.0 μL RNA solution over 5 min by injecting 0.2 μL every minute for 5 min. After injection, the needle was left in the spinal cord for 5 additional min to prevent leakage. After a total of 10 min, the needle was carefully removed and the wound was closed with surgical staples. A similar procedure was used to inject other groups of mice with 500-ng doses of naked mRNA or plasmid DNA loaded in nanomicelles in 1-μL volumes. An additional group of mice was given mRNA-loaded nanomicelles without SCI.

Mice were sacrificed at 48 h post-injury by thoracotomy. Animals were placed on their backs and organs were removed from the thoracic and abdominal cavities to expose the ventral surface of the spinal column. The vertebral bodies were removed using rongeurs to expose the spinal cord. Spinal cords were removed by carefully lifting the tissue from the spinal canal starting from the caudal end toward the rostral end. Spinal nerves were cut with microscissors. Spinal cords were placed in 2 mL cryovials and stored at −80°C until homogenized.

Spinal cord samples were thawed and the injury sites were identified. Approximately 1 cm tissue centered on the injury site was taken and placed into clean 2-mL cryovials with 300 μL 1× Cell Lysis Buffer (Cell Signaling Technology, Danvers, MA, USA). A steel bead was added to each vial, and tissues were homogenized using a Multibeads Shocker (Yasui Kikai, Osaka, Japan). Beads were removed and an additional 1× Cell Lysis Buffer was added to bring volumes to 1 mL each. Samples were centrifuged at 7,000 × *g* for 15 min to remove insoluble debris, and supernatants were transferred to clean 2 mL cryovials and stored at −80°C.

Luciferase activity was measured using a Berthold Lumat LB 9507 single-tube luminometer (Berthold Technologies, Bad Wildbad, Germany). Luciferase Assay System buffer (Promega, Madison, WI, USA) was thawed and prepared according to the manufacturer’s instructions. Each measurement was taken by mixing 100 μL assay buffer with 10 μL tissue lysate that was then immediately placed in the luminometer, with a measurement time of 10 s. Each animal’s sample was measured three times and averaged.

Total protein concentration was determined using a Micro BCA Protein Assay Kit (Thermo Fisher Scientific, Wilmington, DE, USA). Homogenized tissue samples were diluted in PBS at both 100× and 200× so that absorbance values would be more likely to fall within the linear region of the standard curve. Tissue samples were measured in triplicate. After setting up the plate and incubating at 37°C for 2 h, absorbance at 562 nm was determined by Tecan M1000 Pro plate reading spectrophotometer (Tecan, Mannedorf, Switzerland). Protein concentration was determined by linear regression of standard curve absorbance values. Luciferase activity is expressed as Log of relative light unit (RLU) per microgram total protein. Statistical significance was determined by one-way ANOVA followed by Tukey’s multiple comparisons test, performed using GraphPad Prism 7.0a for Mac operating system (OS) X.

### Intraspinal Injection of Luciferase mRNA and BLI

Female C57BL/6J mice (Charles River Laboratories Japan, Yokohama, Japan) (n = 6) were anesthetized by i.p. injection of 0.3 mg/kg medetomidine HCL, 4 mg/kg midazolam, and 5 mg/kg butorphenol tartrate mixture. The spine was surgically exposed under a surgical microscope and stabilized using clamps. Laminectomies were performed at the 11^th^ thoracic vertebra to expose the spinal cord. A 30G needle was carefully used to create an incision in the dura mater. A 500 ng/μL solution of *FLuc* mRNA loaded in nanomicelles was loaded into a glass syringe with a 30G needle. A stereotactic injection apparatus was used to hold the syringe during injection. The needle was carefully inserted into the spinal cord through the previously created dura mater incision, at an angle of 40° from vertical to a depth of 1.5 mm below the surface of the spinal cord. Mice were injected with 1.0 μL RNA solution over 5 min by injecting 0.2 μL every minute for 5 min. After injection, the needle was left in the spinal cord for 5 min to prevent leakage. After a total of 10 min, the needle was carefully removed and the wound was closed with sutures, because surgical staples interfere with BLI. After all data were collected, mice were euthanized by cervical dislocation.

Luciferase expression was measured using the IVIS BLI system (PerkinElmer, Waltham, MA, USA). At 3, 6, 24, 48, 72, 96, 120, 144, 168, and 192 h post-surgery, mice were given i.p. injections of 200 μL 15 mg/mL luciferin (Sumisho Pharmaceuticals International, Tokyo, Japan), and images were taken at 5 min after luciferin injection. Bioluminescence values were log transformed before calculating mean and SD. The limit of detection was determined by multiplying the background SD by 3. The background value was subtracted before data were plotted.

### Contusion SCI and *BDNF* mRNA Injection

Female C57BL/6J mice (n = 8 for BMS experiment, n = 12 for CatWalk experiment) were anesthetized, and their spines were surgically exposed as above. Because the severity of SCI might vary significantly with injury position, the 11^th^ thoracic (T-11) vertebra needed to be reliably identified. This was done by identifying two vertebrae likely to be T-11, then carefully inserting a 26G needle under the supraspinal ligament between them. Mice were transferred to a CosmoScan GX micro-computed tomography (CT) instrument (Rigaku, Tokyo, Japan) and imaged. The needle is easily observed on the CT image, and the vertebra position can be confirmed by counting the ribs, assuming that the last set of ribs is on vertebra T-13. Once the correct vertebra was identified, the spinal column was restrained with forceps and laminectomy was performed.

After laminectomy, the restrained mouse was moved to an Infinite Horizons IH-0400 Impactor. The impactor tip was carefully aligned with the center of the exposed spinal cord surface and lowered until the tip barely touched the surface. The tip was then raised by turning the vertical adjustment knob 4 turns, approximately 5 mm above the spinal cord surface. Impacts were carried out using a peak impact force of 50 kdyne.

After impact, mice in the *BDNF* RNA or DNA groups were moved to a stereotactic injection apparatus, and 500 ng *BDNF* mRNA loaded in nanomicelles was injected into the injured spinal cord as described above. After injection, the incisions were closed using surgical staples. Mice in the untreated group were not given injections, the incisions were closed immediately after injury.

Mice were carefully monitored after injury. Bladders were manually pressed daily to drain the urine and prevent urinary tract infections. Motor function was measured by the BMS or CatWalk Automated Gait Analysis System, as described below for 6 weeks. After all measurements were taken, mice were euthanized by cervical dislocation.

### BMS Measurements

Mice were placed in a 30 × 30 × 15-cm plastic cage and visually observed for 5 min. A checklist of behavioral patterns (ankle movement, weight support, plantar stepping, etc.) was used to determine BMS score and subscore.^30^ Statistical significance was determined by 2-tailed t test between injured mice with *BDNF* mRNA and injured mice without *BDNF* mRNA. Observations were not carried out in a blinded manner.

### CatWalk Automated Gait Analysis System

The CatWalk XT Automated Gait Analysis System (Noldus Information Technology, Wageningen, the Netherlands) is an instrument with a glass platform above a high-resolution camera. Green light is internally reflected inside the glass platform, and a mouse is allowed to walk on the platform. Light is reflected downward toward the camera wherever the mouse contacts the glass, and higher pressures create higher intensities of reflected light. The video is analyzed by software, and several parameters describing the animal’s gait are calculated.^31^, ^41^

Injured mice were measured on the CatWalk XT instrument every week from 1 to 6 weeks post-injury. Runs were considered compliant if the animal crossed the camera’s 30-cm field of view in less than 12.5 s and did not turn around during the run. Three compliant runs were collected for every mouse at every time point and combined into a trial. However, poorly performing mice were often not able to complete a run in less than 12.5 s. In these cases, runs with durations above 12.5 s were used. Non-injured mice were used as controls.

Run videos must be classified to identify the animal’s paws before gait analysis can be performed. The CatWalk XT software has an automated classification tool, but this tool has a significant error rate. Videos were classified by using the automated classification tool, then they were carefully manually checked to correct mistakes. After classification, trial statistics were calculated and exported as an Excel spreadsheet file. Data were analyzed using a custom-written C++ program and plotted using Gnuplot (see the Supplemental Materials and Methods for more information).

### Combined CatWalk Index Scoring

Because the CatWalk instrument produces such a large number of parameters, a method to combine all of the data into a single score was used. Briefly, data from BMS scores were correlated with CatWalk data to determine least-squares linear regression coefficients for each parameter. These regression values were combined in a weighted average, where the R^2^ value was used as the weighting value, so that strongly correlated parameters would be given strong weights and weakly correlated parameters would be given weak weights. This method is referred to as the combined CatWalk index.^32^ The combined CatWalk index scores were calculated from the CatWalk data using the previously published method. Statistical significance was determined by one-way ANOVA, followed by Tukey’s test for multiple comparisons.

### BDNF Expression Measurement

BDNF expression was determined by ELISA. Female C57BL/6J mice (n = 3) were anesthetized, injured, and injected with *BDNF* mRNA nanomicelles, as described above. Mice were euthanized by thoracotomy, and spinal cord tissue was collected and homogenized as above.

BDNF concentrations were determined using the BDNF E_max_ ImmunoAssay System (Promega, Madison, WI, USA). Briefly, 96-well Costar ELISA plates (Corning, Oneonta, NY, USA) were coated with anti-BDNF monoclonal antibodies in carbonate buffer at 4°C overnight. Coated plates were blocked the next day. Homogenized tissue samples were diluted by mixing 50 μL tissue samples with 200 μL blocking buffer. (The manufacturer’s protocol suggests acidifying and neutralizing the homogenized tissue samples to improve BDNF measurements and that this effect was tissue and species specific. In this study, acid treatment was observed to reduce measured BDNF concentrations by a factor of 10; therefore, acid treatment was not performed in subsequent ELISAs.) Samples were added to the top row of the blocked plate along with BDNF standards. A 2:1 serial dilution was performed down the plate. After incubation with BDNF solutions, anti-BDNF polyclonal antibodies and then horseradish peroxidase (HRP)-conjugated secondary antibodies were added. TMB-One solution was used for color development, and reactions were stopped with 1 N HCl. Absorption at 450 nm was determined using a TECAN Infinite M1000 Pro plate reading spectrophotometer. BDNF concentration was determined by linear regression of standard curve absorbance values. Non-injured mice, injured mice without mRNA nanomicelles, and injured mice with *BDNF* mRNA nanomicelles were tested, with group sizes of 3 mice each.

Total protein concentrations were determined by bicinchoninic acid (BCA) assay as before. BDNF concentrations are reported as picograms BDNF per microgram total protein. Statistical significance was determined by one-way ANOVA followed by Tukey’s multiple comparisons test, using GraphPad Prism 7.0a for Mac OS X (GraphPad, La Jolla, CA, USA).

### Luxol Fast Blue Staining

Female C57BL/6J mice (n = 7) were anesthetized, and laminectomies, contusion SCI, and *BDNF* mRNA nanomicelle injection were performed as above. At 2 weeks post-injury, mice were sacrificed by transcardial perfusion with 10 mL PBS followed by 10 mL 4% paraformaldehyde. Spinal cords were removed and placed in 10-mL solutions of 4% paraformaldehyde and placed on ice. Samples were embedded in paraffin and cut into transverse sections of 4-μm thickness at 0, 200, and 400 μm from the injury site, then stained with Luxol Fast Blue. Microscope images were obtained using a Keyence All-In-One BZ-X700 Microscope (Keyence, Itasca, IL, USA). Images were analyzed by dividing the images into myelinated and nonmyelinated portions, and the area of each was measured using ImageJ. Statistical significance was determined by one-way ANOVA followed by Tukey’s multiple comparisons test, using GraphPad Prism 7.0a for Mac OS X (GraphPad, La Jolla, CA, USA).

### Immunostaining

Female C57BL/6J mice (n = 6) were anesthetized, and laminectomies and contusion SCI were performed as above. Spinal cords were injected with 500 ng *BDNF* mRNA or *BDNF* DNA loaded in nanomicelles, or they were not injected as a non-treated control. At 24 h post-injection, mice were anesthetized again and sacrificed by transcardial perfusion with 10 mL PBS followed by 10 mL 4% paraformaldehyde. Spinal cords were removed and placed in 10-mL solutions of 4% paraformaldehyde. Samples were embedded in paraffin and cut into transverse sections of 4-μm thickness.

Spinal cord sections were blocked with donkey fragment antigen binding (F(ab)) fragment anti-mouse immunoglobulin G (IgG) H&L antibody (Abcam, Cambridge, UK), and then they were stained with the following primary antibodies: anti-BDNF (1:500, rabbit monoclonal, Abcam, Cambridge, UK), and anti-APC (1:50, mouse monoclonal, Abcam, Cambridge, UK). Sections were then stained with secondary antibodies conjugated with Alexa Fluor 488 or Alexa Fluor 546 (a:200, Invitrogen, Carlsbad, CA, USA). After staining with DAPI (Thermo Fisher Scientific, Waltham, MA, USA), the sections were observed with a Keyence All-In-One BZ-X700 microscope.

BDNF expression was analyzed by measuring the 545-nm excited fluorescence intensity of APC-positive cells within a 0.175-mm^2^ (500 × 350-μm) area of gray matter using the Hybrid Cell Count Module BZ-H3C software (Keyence, Itasca, IL, USA). Statistical significance was determined by one-way ANOVA followed by Tukey’s multiple comparisons test, using GraphPad Prism 7.0a for Mac OS X (GraphPad, La Jolla, CA, USA).

### Inflammatory Cytokine Expression

Inflammatory cytokine expression was determined using qRT-PCR. Female C57BL/6J mice (n = 5) were given either no mRNA or *BDNF* mRNA nanomicelles. The no injury-no RNA control group was given laminectomy, but not injections or contusion SCI. Other groups were given laminectomy and contusion SCI and/or mRNA injection as appropriate. RNA-treated mice were given 500-ng doses of *BDNF* mRNA-loaded nanomicelles immediately after injury.

Spinal cord tissue was collected at 48 h post-injury as before, but samples were placed in 2-mL cryovials, flash frozen in liquid nitrogen, and stored at −80°C. 300 μL Qiazol (QIAGEN, Hilden, Germany) was added to each tissue sample, and samples were homogenized and centrifuged as before. Supernatants were transferred to clean vials, and 100 μL chloroform and 300 μL H_2_O were added to each sample. Samples were mixed well and centrifuged at 12,000 × *g* for 15 min. Aqueous phases were transferred to clean vials and 600 μL isopropanol was added to each. Samples were mixed well, allowed to sit for 10 min, then centrifuged for 10 min to precipitate RNA. RNA pellets were washed with 500 μL 75% ethanol and centrifuged for 5 min.

RNA pellets were resuspended in 88 μL RNase-free H_2_O, and genomic DNA was removed using the QIAGEN RNase Free DNase Set (QIAGEN, Hilden, Germany); RNA was purified using the QIAGEN RNeasy kit, according to the manufacturer’s instructions for RNA cleanup. RNA was eluted using 100 μL RNase-free H_2_O. RNA quantity and quality were determined using both Nanodrop 2000 and Bioanalyzer instruments. cDNA was synthesized using the Toyobo cDNA Synthesis kit (Toyobo, Osaka, Japan). Each cDNA reaction used 120 ng RNA to reduce variability due to different amounts of RNA.

qPCR was performed using the following Taqman assays: Mouse Actin (MOUSE ACTB[DQ]), TNF-α (Mm00443258), IL-1β (Mm00434228), IL-6 (Mm00446190), IL-4 (Mm00445259), and IL-10 (Mm01288386) (Applied Biosystems, Waltham, MA, USA), using a Bio-Rad CFX Connect Real-Time PCR System. PCR-cycling conditions were 95°C for 20 s followed by 40 cycles of 95°C for 3 s and 60°C for 30 s. Each sample was measured in triplicate. Data were analyzed using the ΔΔC_t_ method to determine expression relative to actin control and the no injury-no RNA group. Statistical significance was determined by one-way ANOVA followed by Tukey’s multiple comparisons test, using GraphPad Prism 7.0a for Mac OS X (GraphPad, La Jolla, CA, USA).

## Author Contributions

S.T.C. designed and conducted the experiments and wrote the manuscript. Y.F. performed tissue sectioning and immunofluorescent staining. Y.F., S.U., K.K., and K.I. contributed to study design. All authors read and approved the final manuscript.

## Conflicts of Interest

The authors declare no competing interests.

## Supplemental Information

Document S1. Figures S1–S11 and Supplemental Materials and Methods

Document S2. Article plus Supplemental Information
