# Supplementary material for: Enhancement of Motor Function Recovery after Spinal Cord Injury in Mice by Delivery of Brain-Derived Neurotrophic Factor mRNA
Source: Mol Ther Nucleic Acids. 2019 Jun 29;17:465–76. doi: 10.1016/j.omtn.2019.06.016 (PMC6658833; doi:10.1016/j.omtn.2019.06.016)
Supplement: Document S1. Figures S1–S11 and Supplemental Materials and Methods [file mmc1.pdf]

OMTN, Volume *17*

Supplemental Information

Enhancement of Motor Function Recovery after Spinal Cord Injury in Mice by Delivery of Brain-Derived Neurotrophic Factor mRNA

Samuel T. Crowley, Yuta Fukushima, Satoshi Uchida, Kazunori Kataoka, and Keiji Itaka

Supplementary Figures

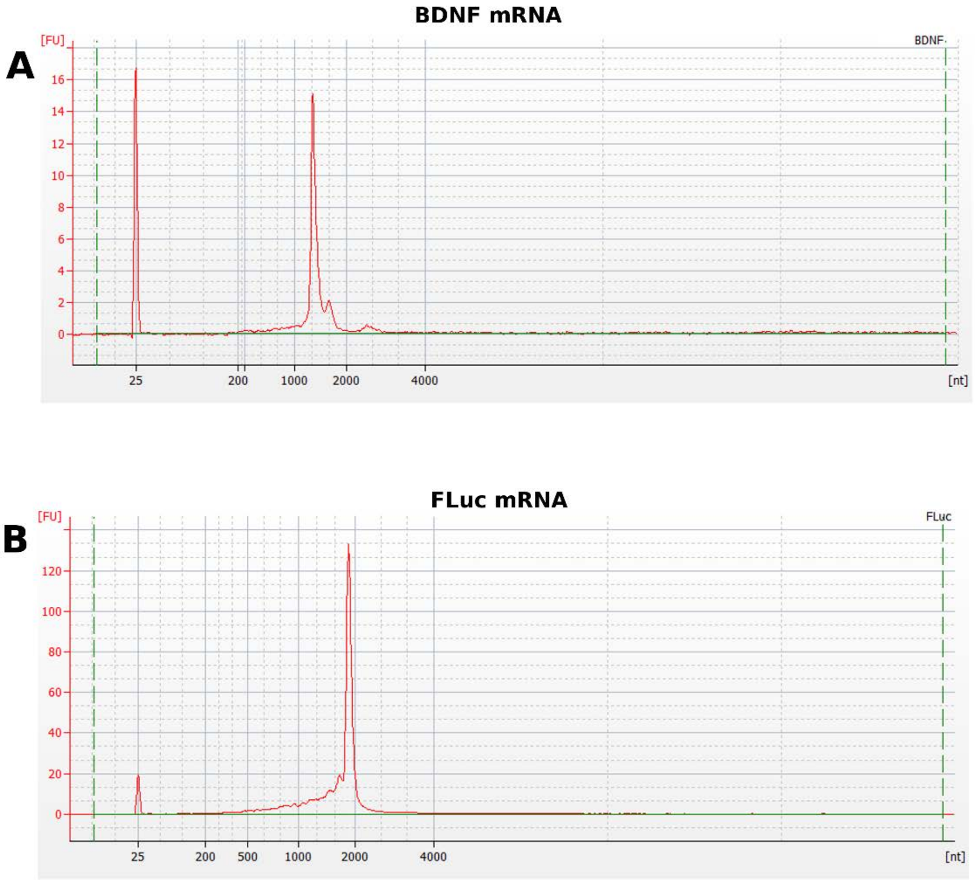

Supplementary Figure 1: Bioanalyzer Analysis of mRNA. BDNF mRNA and FLuc mRNA were analyzed by Agilent Bioanalyzer in **A** and **B** respectively.

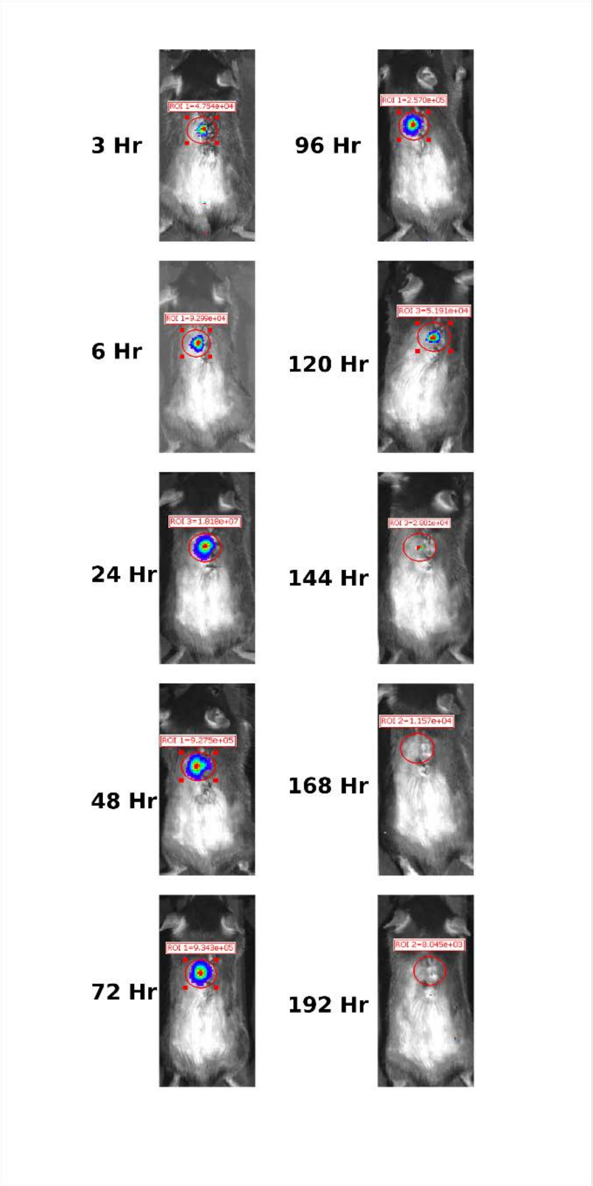

Supplementary Figure 2: Representative BLI images. Images of the mouse from Figure 1C at each time point.

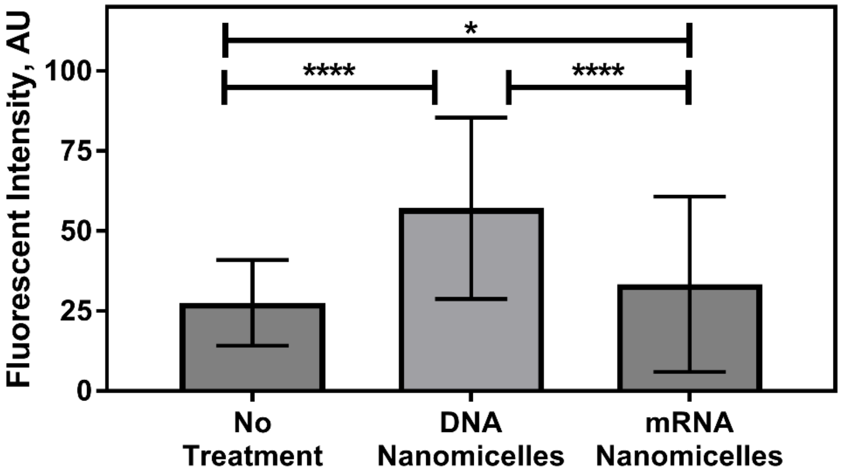

Supplementary Figure 3: Quantification of BDNF expression from immunofluorescence data. Spinal cord sections were stained with anti-BDNF antibodies and fluorescence of each cell was measured. All error bars represent standard deviation.

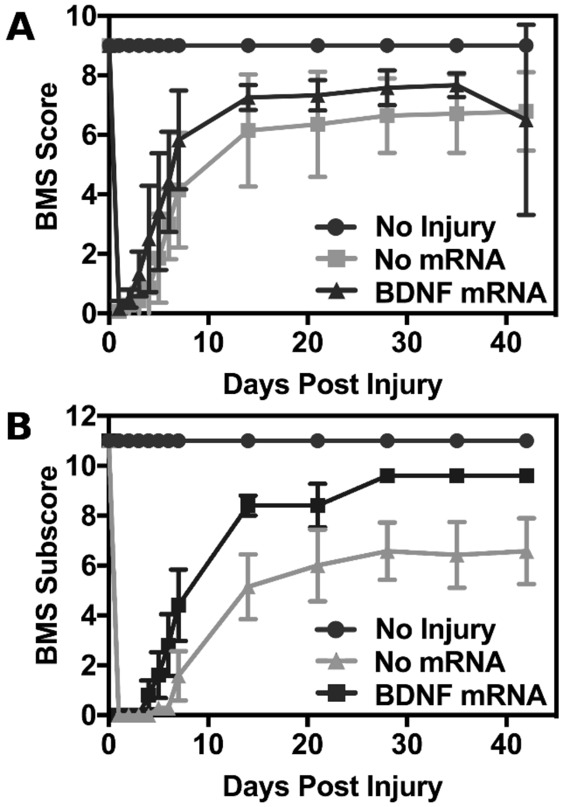

Supplementary Figure 4: Basso Mouse Scale assessment of Motor Function. Mice were given contusion SCI with peak impact forces of 50 kdynes and immediately injected with 500 ng doses of

BDNF mRNA loaded in nanomicelles directly at the site of injury. Mice were monitored over 6 weeks using the Basso Mouse Scale to assess motor function recovery. BMS Scores are shown in **A**, while Subscores are shown in **B**. All error bars represent sample standard deviation.

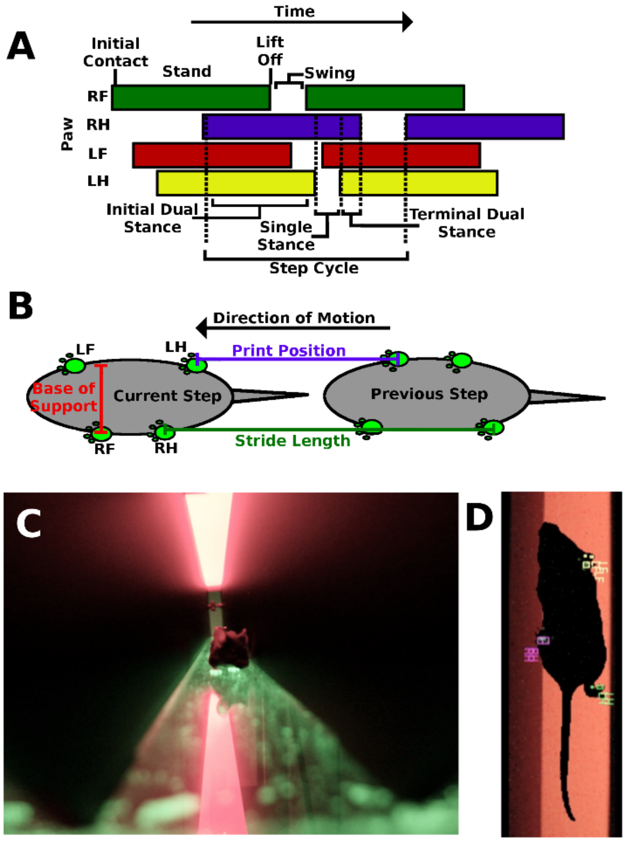

Supplementary Figure 5: CatWalk Automated Gait Analysis System. The CatWalk instrument collects video of mice as they walk across a glass platform and analyzes the video to determine several parameters related to their gait. Many of these parameters are derived from the timing of how paws are placed on the glass, shown in **A**. The four paws are identified as Right Front, Right Hind, Left Front, and Left Hind (**RF**, **RH**, **LF**, and **LH**). The time when a paw is on the glass is marked with a colored rectangle, and is called the Stand time. The time when a paw is off the glass is called the Swing time. The total time between steps is the Step Cycle, and is the sum of Stand and Swing. Initial Dual Stance, Single Stance, and Terminal Dual Stance are determined from the time a paw is on the glass at the same time its contralateral paw is on the glass. In this case, the values for the RH paw

are determined based on overlapping times with the LH paw. Other parameters are determined based on paw position, as shown in **B**. As the mouse walks, the distance a paw moves between steps is called the Stride Length, the distance between contralateral paws is called the Base of Support, and the distance between a paw on the current step and its ipsilateral paw on the previous step is called the Print Position. The images in **C** and **D** show a mouse walking on the CatWalk platform during a measurement. The green light used to identify the paw positions and the orange overhead light used to create a silhouette of the mouse body are clearly seen in **C**. A frame from the recorded and classified video is shown in **D**, three paws are on the glass and labeled **LF**, **LH**, and **RH**.

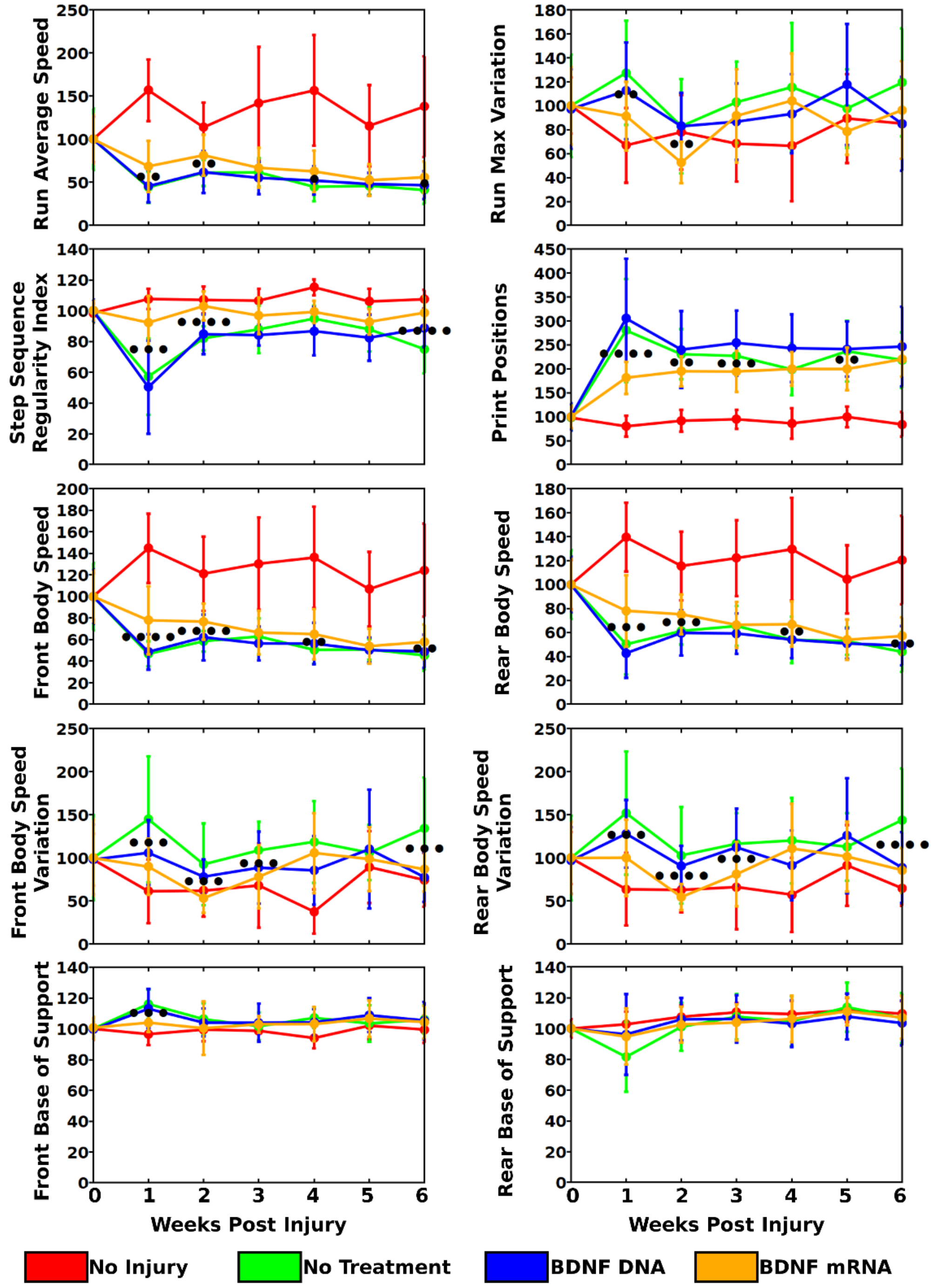

Supplementary Figure 6: CatWalk data related to general motor function measurements, including speed and variation in speed, Step Sequence Regularity Index, Print Positions, and Base of Support. RNA treated mice show significantly different speed and coordination than their non-treated counterparts.

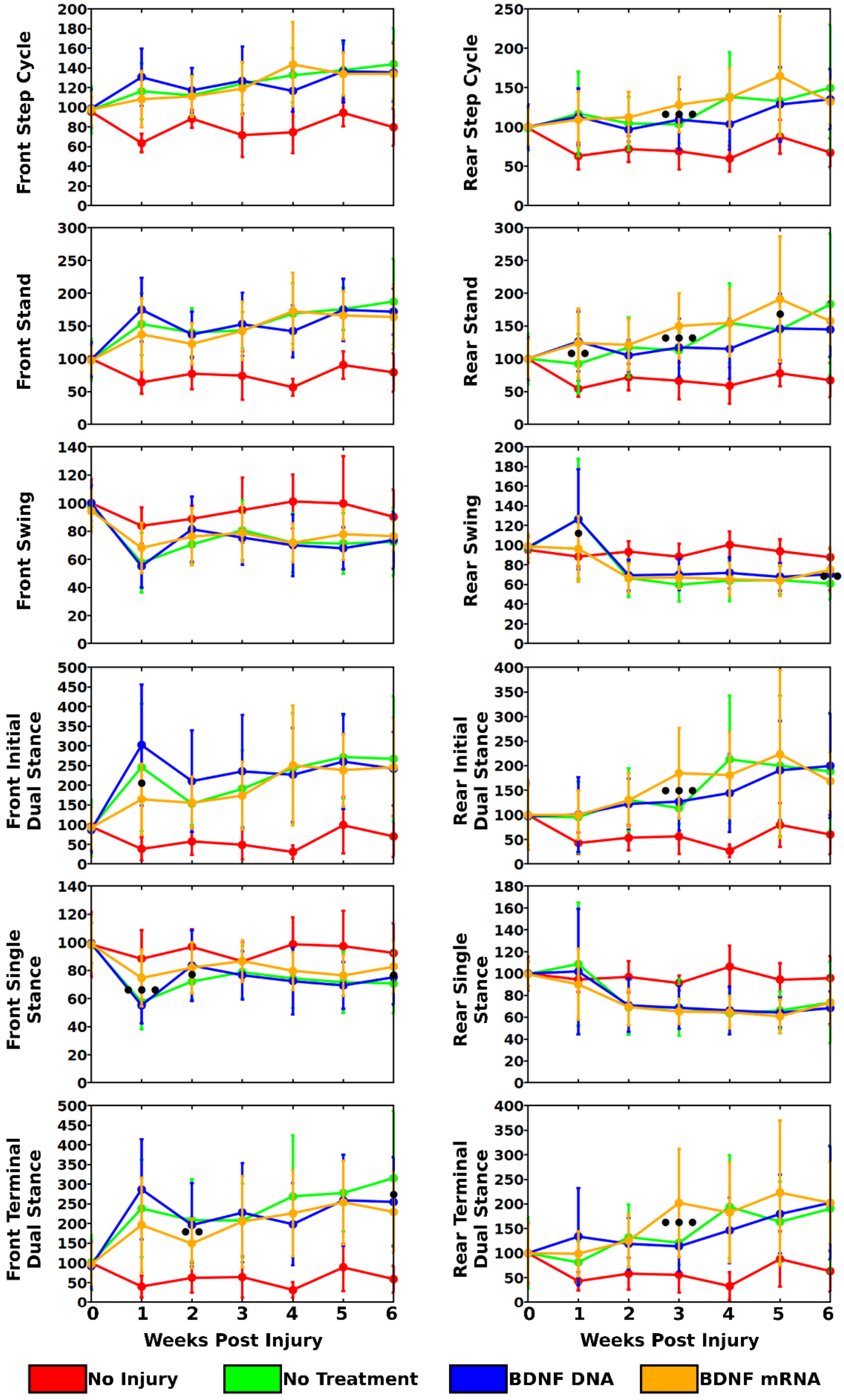

Supplementary Figure 7: CatWalk data related to Step Cycle timing, including Step Cycle, Stand, Swing, Initial Dual, Single, and Terminal Dual Stance times.

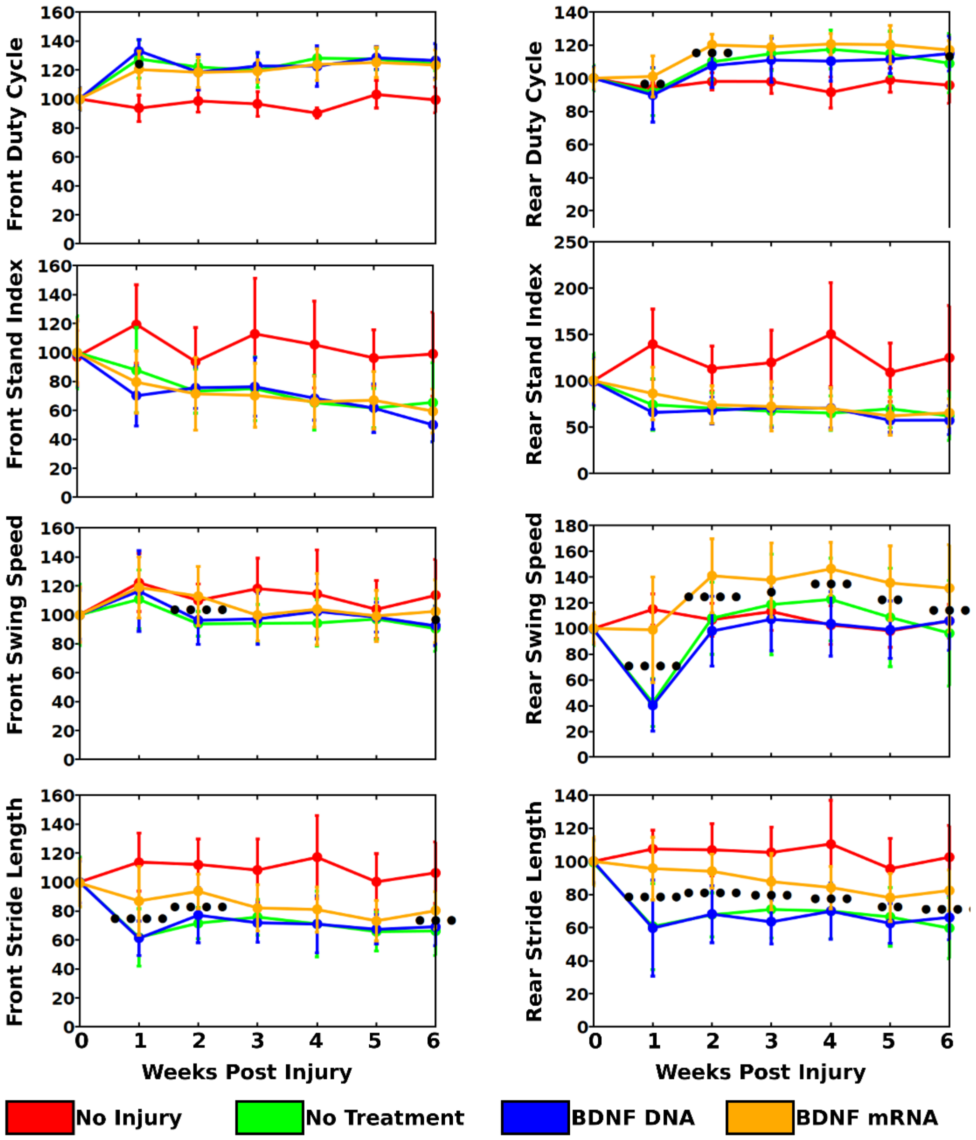

Supplementary Figure 8: CatWalk measurements derived from Step Cycle timing measurements. RNA-treated mice show significantly different Front Duty Cycle, Rear Swing Speed, and Front and Rear Stride Lengths.

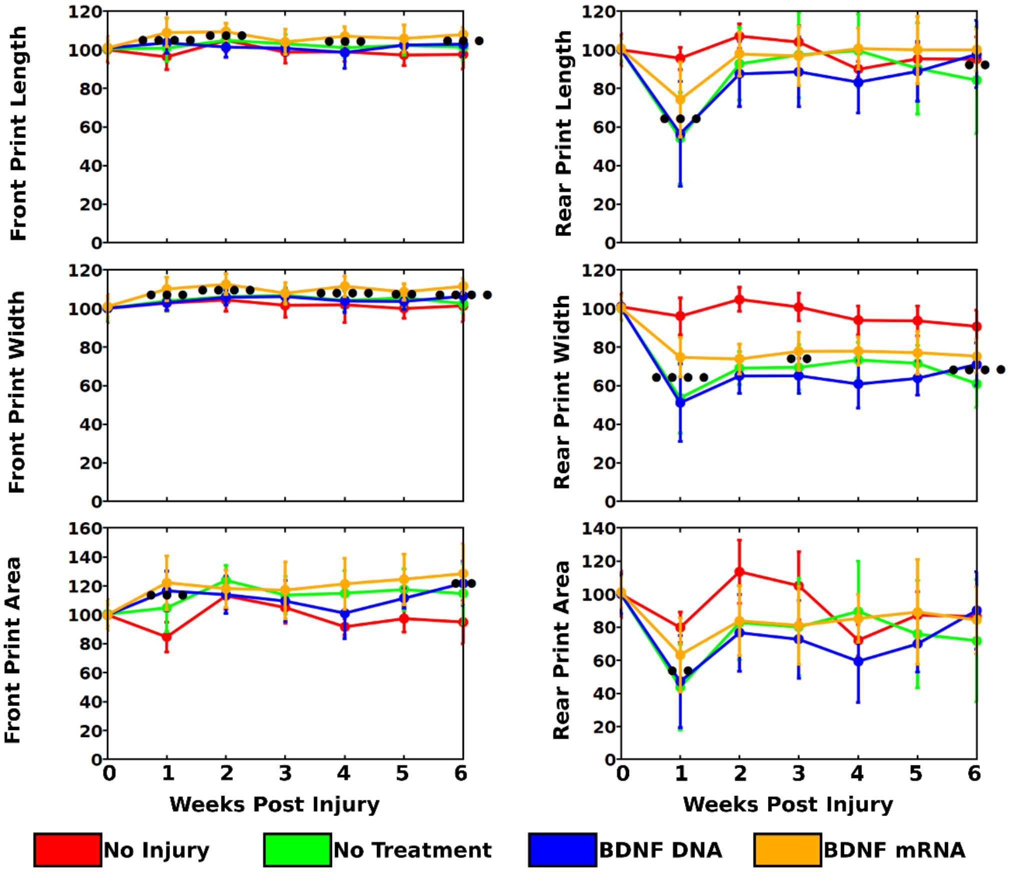

Supplementary Figure 9: CatWalk measurements related to paw size.

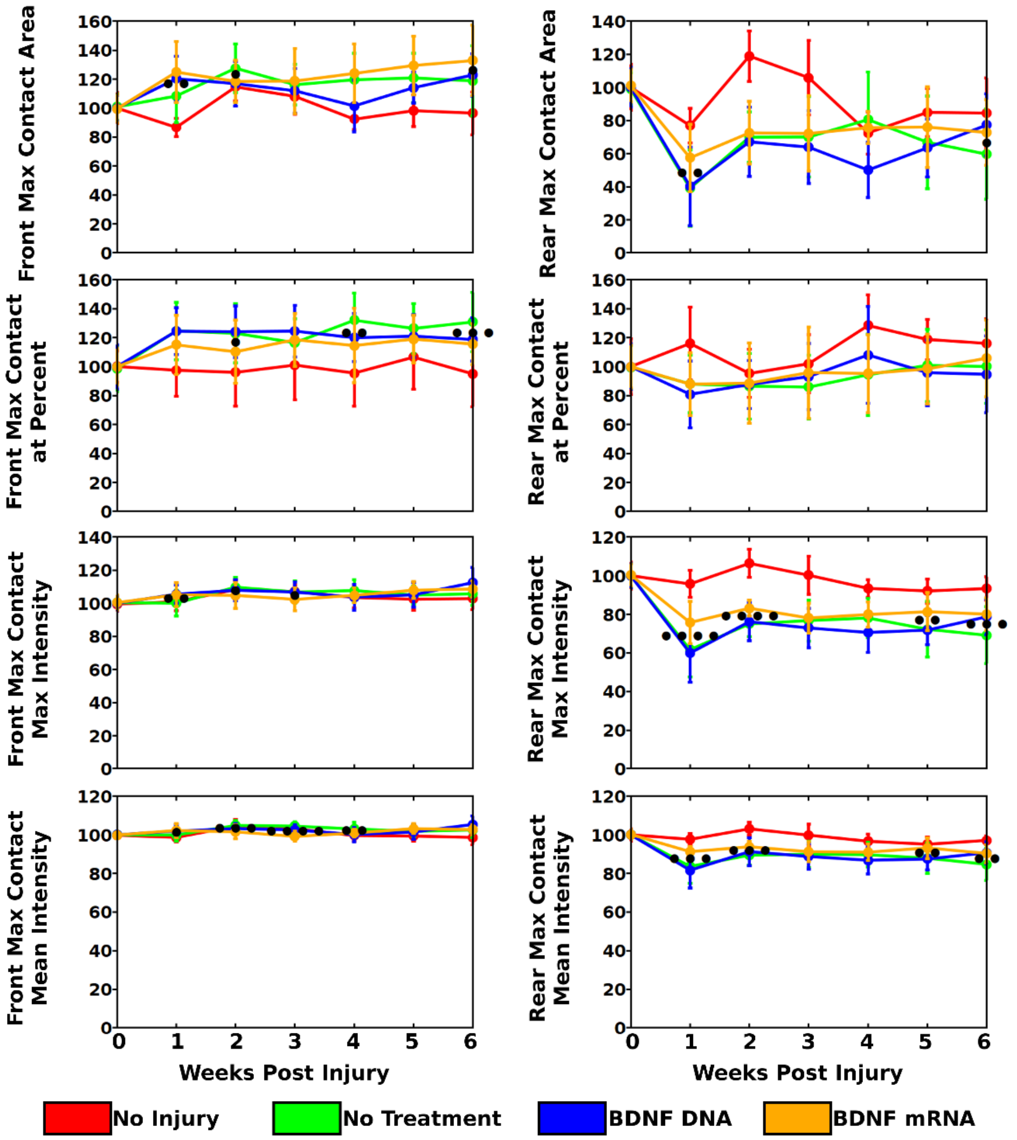

Supplementary Figure 10: CatWalk measurements related to contact area and intensity at the time of maximum contact.

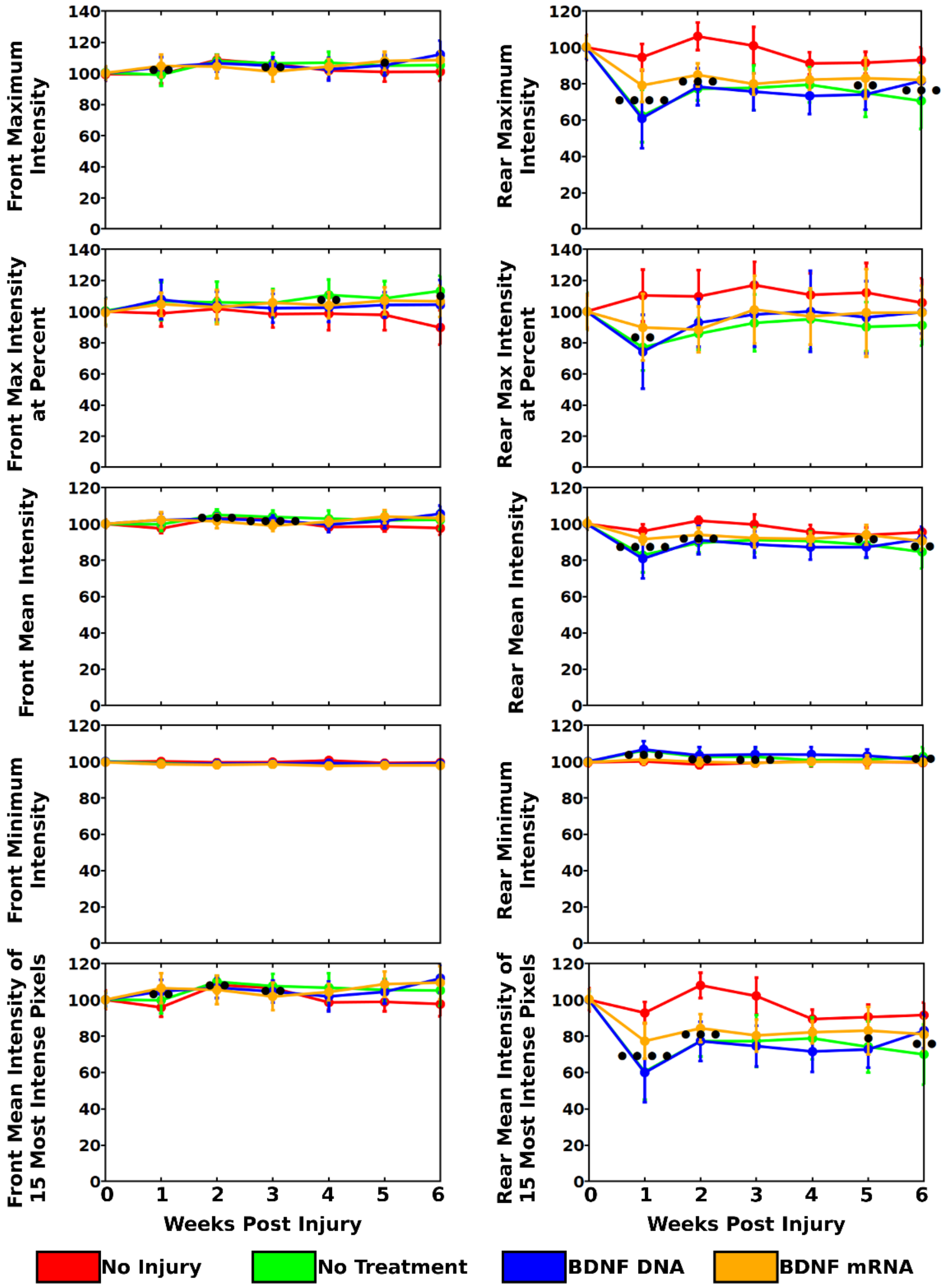

Supplementary Figure 11: CatWalk measurements related to intensity. *BDNF* mRNA treated mice have significantly higher maximum and mean rear intensity, and significantly lower minimum intensity than non-treated mice.

Supplementary Material

Explanation of CatWalk Terms

The Noldus CatWalk Automated Gait Analysis System produces a large amount of data, with several different parameters. Several of the parameters are Step Cycle measurements (**Supplementary Figure 5A**). The Step Cycle is the time in seconds between placing a paw on the glass platform and placing the paw back on the glass for the next step. The Step Cycle is divided into 2 portions, the Stand and Swing. The Stand measurement is the amount of time the paw stays on the glass, while the Swing measurement is the amount of time the paw is lifted off the glass between steps. Additional measurements are determined from the times that a paw is on the glass while the contralateral paw is also on the glass. For example, if the Right Hind paw is placed down while the Left Hind paw is already on the glass, the time where both paws are on the glass is called the Initial Dual Stance. When the Left Hind paw is lifted off the glass, leaving the Right Hind paw on the glass, this time is called the Single Stance. When the Left Hind paw is placed back on the glass before the Right Hind paw is lifted off the glass, this is called the Terminal Dual Stance.

Other measurements are determined from the positions of the paws during each step (**Supplementary Figure 5B**). The Stride Length is the distance a single paw moves between steps. The Base of Support is the distance between the Left and Right Paws, and is calculated for both the Front and Hind paws. The Print Position measurement is the distance between a paw's current position and the position of the ipsilateral paw during the previous step.

Other parameters are derived from the above parameters, such as the Duty Cycle, which is the Stand time divided by the Step Cycle time. The Swing Speed is the Stride Length divided by the Swing time. Additionally, each pawprint is individually analyzed to determine Print Length, Width, and Area. The print area changes during the Stand time as the paw touches the glass, weight is applied, and then pushes the animal forward. The area is monitored over time, and the Maximum Contact Area for each step is identified, as well as the time that maximum contact occurs, which is divided by Stand Time to create Maximum Contact at Percent. Intensity at Maximum Contact are

also determined as Max Contact Max Intensity and Max Contact Mean Intensity. However, maximum intensity can occur at a time different from Max Contact Area, so the time at Maximum Intensity is divided by Stand time to determine Max Intensity at Percent.

Coordination is measured by keeping track of the order in which each paw is placed. Healthy mice place their paws in one of 6 regular patterns, but injured mice tend to have a more random pattern. The percent of steps falling into one of the regular patterns is determined as the Step Sequence Regularity Index.

CatWalk Data Analysis Software

Because the CatWalk system produces a large amount of data, it is difficult to manually analyze the data. Custom software was written in C++ to assist in data analysis. The software takes the data from the spreadsheet and sorts it into individual mice and treatment groups, and pools data from left and right limbs. Data from pre-injury baseline measurements are averaged, and data is presented as a percentage of these baseline values. Quartiles are determined and outliers are removed by Tukey's Fences method using a K value of 1.5. After outliers are removed, quartiles are recalculated, and mean, median, maximum, minimum, sample standard deviation, and standard error of the means are calculated. Statistical significance is determined using a two-tailed unpaired Welch's T Test, which does not assume equal sample sizes or variances. Because Welch's T Test can produce non-integer degrees of freedom, critical T values are determined by linear interpolation of T values with the nearest integer degrees of freedom. Statistical significance is reported according to confidence level, •: $P < 0.10$, ••: $P < 0.05$, •••: $P < 0.01$, ••••:$P < 0.001$. The data is then passed to gnuplot for drawing graphs.
